# Malaria Attributable to the HIV-1 Epidemic, Sub-Saharan Africa

**DOI:** 10.3201/eid1109.050337

**Published:** 2005-09

**Authors:** Eline L. Korenromp, Brian G. Williams, Sake J. de Vlas, Eleanor Gouws, Charles F. Gilks, Peter D. Ghys, Bernard L. Nahlen

**Affiliations:** * World Health Organization, Geneva, Switzerland;; † Erasmus University Medical Center, Rotterdam, the Netherlands;; ‡ Joint United Nations Programme on HIV/AIDS, Geneva, Switzerland

**Keywords:** malaria, epidemiology, death, illness, prevention and control, HIV-1 complications, opportunistic infections, Africa, time trends, research

## Abstract

We assessed the impact of HIV-1 on malaria in the sub-Saharan African population. Relative risks for malaria in HIV-infected persons, derived from literature review, were applied to the HIV-infected population in each country, by age group, stratum of CD4 cell count, and urban versus rural residence. Distributions of CD4 counts among HIV-infected persons were modeled assuming a linear decline in CD4 after seroconversion. Averaged across 41 countries, the impact of HIV-1 was limited (although quantitatively uncertain) because of the different geographic distributions and contrasting age patterns of the 2 diseases. However, in Botswana, Zimbabwe, Swaziland, South Africa, and Namibia, the incidence of clinical malaria increased by <28% (95% confidence interval [CI] 14%–47%) and death increased by <114% (95% CI 37%–188%). These effects were due to high HIV-1 prevalence in rural areas and the locally unstable nature of malaria transmission that results in a high proportion of adult cases.

## Abstract 2

The HIV-1 epidemic has increased the malaria disease and death rate in southern Africa.

HIV-1 infection increases the risk and severity of malaria (*1**,**2*). In African settings of both high- and low-intensity (epidemic, unstable, or strongly seasonal) malaria transmission, increases in the severity and case fatality of malaria have been observed in all age groups (*3**–**8*). In areas of high-intensity transmission, HIV-1 also increases the incidence of clinical malaria among adults (*9*).

The population-level impact depends on HIV prevalence, the age distribution of both infections (which for malaria is determined by the transmission intensity), and their geographic overlap. Local distributions of CD4 cell counts and clinical stages of HIV-infected patients are also important because the effects of HIV multiply with increasing immunosuppression (*9**,**10*).

The HIV-1 epidemic in Africa has matured over the past 25 years and may now be reaching a peak (*11*). From the 1980s to the 1990s, malaria death and disease increased, especially among children in rural, malaria-endemic parts of East and West Africa (*12*), and in populations at risk for unstable malaria in South Africa (*13**–**15*). Chloroquine resistance (*16*), breakdown of vector-control operations (*17*), and HIV-1 may all have contributed to these trends (*3**,**4**,**13*). We assessed the number of additional malaria cases and deaths caused by HIV-1 in sub-Saharan Africa by calculating the impact of HIV-1 separately for urban and rural populations, areas of high- and low-intensity malaria transmission, and different age groups in each country.

## Methods

### Malaria Incidence in the Absence of HIV-1

In the absence of reliable data from routine health information systems, the incidence of uncomplicated and severe clinical malaria episodes, collectively referred to here as "malaria incidence," was calculated from estimates of incidence rates (Table 1) and national populations at risk for malaria in 2004 (Table 2), by using the 1998 map of climatic suitability for malaria transmission (*18*). The climate suitability index was used to indicate high- (>0.75) or low-intensity transmission (>0 and <0.75) (*19*). For high-intensity transmission areas, average incidence of malarial fevers in the absence of HIV was estimated at 1.4 per person per year among children <5 years of age, 0.59 per person per year in those 5–14 years of age, and 0.11 per person per year in those >15 years of age (*19*). In areas of low transmission, incidence among those <5 years of age was estimated at 0.182 per person per year (*19*). Because immunity against clinical malaria increases slowly with age in areas of low-intensity transmission, the incidence rate among 5- to 14-year-olds and those >15 years were considered to be the same as that in young children and half that rate, respectively (*19*).

**Table 1:** HIV-1, malaria incidence and death rates. and their interactions*

Parameter	Assumption
Malaria transmission intensity	Index >0 and <0.75 denotes low-intensity transmission and >0.75 denotes high-intensity transmission, except for southern Africa, where index >0.75 denotes unstable transmission (*18**,**19*)
Overall malaria incidence	Middle Africa, high-transmission areas: 1.4 per person per year in children <5 y, 0.59 per person per year at 5–14 y, 0.11 per person per year at >15 y (*19*) Middle Africa, low-transmission areas: 0.182 per person per year in children <15 y, 0.091 per person per year at >15 y (*19*) Southern Africa: 0.0294 per person per year as all-age average in areas with (unstable) malaria transmission; divided as twice the rate at >15 y compared to <14 y (*19*)
Relative malaria incidence urban/rural	0.50 (20)
Malaria deaths	High-transmission areas: 0.8% of incident cases in children <5 y, 0.3% at >5 y; Low-transmission and unstable transmission areas: 0.8% of incident cases in all age groups (*21*).
Effect of HIV-1 on incidence of clinical malaria	>5 years in areas with high-intensity malaria transmission, and all age groups in areas with low-intensity or unstable malaria transmission: CD4 >500/μL RR = 1.2 CD4 200–499/μL RR = 3.0 CD4 <200/μL RR = 5.0† <5 years in high-transmission areas: no effect
Effect of HIV-1 on malaria case fatality rate	All malaria transmission intensities and age groups: CD4 >500/μL RR = 2.0 CD4 200–499/μL RR = 4.0 CD4 <200/μL RR = 10‡
Survival after HIV-1 infection	Median 9 years, following a Weibull curve with shape parameter 2.28 (*22*)
CD4 decline over the course of HIV-1 infection	Linear from 825/μL at seroconversion to 20/μL at death of AIDS (*23**–**26*)

*RR = relative risk associated with HIV-1 infection; y = years of age. †From (*9**,**10*). Earlier studies did not consistently show these effects, but these were cross-sectional and/or hospital-based (*1*). Effects of HIV-1 in these studies may have been obscured by a lack of adjustment for prestudy treatment with antimalarial drugs (which might be more common in HIV-1 patients with recurrent fevers [27]) and by their inherent dependence on the relative survival of HIV-infected and HIV-uninfected participants, given the increased case fatality of malaria among HIV-infected patients (*6*). At the specified CD4-stratum-specific relative risks, the relative risk averaged over all HIV-infected people would be 2.1 in Madagascar and 2.5 in all other countries (see Methods, CD4 distributions among HIV-infected people). ‡At these CD4-stratum-specific relative risks, the relative risk averaged over all HIV-infected people would be 3.4 in Madagascar and 4.1 in all other countries (see Methods, CD4 distributions among HIV-infected people).

**Table 2:** Estimated HIV-1 impact on malaria cases and deaths, sub-Saharan Africa, 2004*

Country†	Urban, %	Population at risk for malaria, by intensity of transmission, %		HIV-1 prevalence in adults, %		Malaria incidence/1,000 py‡		Malaria deaths/1,000 py‡	
		Low or unstable	High	National§	Urban-rural ratio¶		Increase due to HIV, %		Increase due to HIV, %
Angola	34	53	46	3.9	1.6#	291	1.2	1.8	4.2
Benin	42	0	100	1.9	1.8	434	0.4	2.4	1.4
Botswana	49	13	0	37.3	1.0	3.5	28.0	0.028	114.4
Burkina Faso	17	0	100	4.2	3.1	538	0.8	3.1	2.9
Burundi	9	64	21	6.0	3.6	209	2.4	1.4	8.6
Cameroon	49	24	74	6.9	1.7	317	1.9	1.8	6.5
Central African Republic	41	0	100	13.5	1.2	419	3.2	2.3	11.3
Chad	24	14	86	4.8	1.3	451	1.1	2.6	4.1
Congo	65	0	100	4.9	1.6#	380	1.1	2.1	3.8
Côte d'Ivoire	44	0	100	7.0	2.1	402	1.6	2.2	5.2
Democratic Republic of the Congo	30	10	85	4.2	1.6	423	0.9	2.4	3.3
Equatorial Guinea	48	2	97	11.6	1.6#	401	2.5	2.3	8.50
Eritrea	19	83	16	2.5	1.6#	197	1.3	1.4	4.2
Ethiopia	16	50	14	4.4	4.8	142	1.7	1.0	5.9
Gabon	81	0	96	8.1	1.9	287	1.9	1.5	6.0
Gambia	31	0	100	1.2	0.7	429	0.3	2.3	1.0
Ghana	36	2	98	3.1	1.2	401	0.8	2.1	2.6
Guinea	28	1	99	3.2	1.6#	468	0.7	2.6	2.2
Guinea-Bissau	32	0	100	3.8	1.6#	480	0.7	2.7	2.7
Kenya	33	57	21	6.7	1.8	164	2.9	1.1	10.4
Liberia	45	0	100	5.9	1.6#	437	1.1	2.5	4.0
Madagascar	30	36	60	1.7	0.7	327	0.4	1.9	1.3
Malawi	15	22	77	14.2	1.6#	435	3.5	2.6	13.3
Mali	30	10	90	1.9	1.5	464	0.4	2.7	1.5
Mauritania	58	59	41	0.6	1.6#	221	0.2	1.4	0.72
Mozambique	32	4	96	12.2	1.2	437	2.8	2.4	10.3
Namibia	31	8	0	21.3	1.3	2.3	14.5	0.018	52.4
Niger	21	11	89	1.2	3.2	496	0.2	2.9	0.8
Nigeria	44	1	99	5.4	1.1	420	1.2	2.3	4.3
Rwanda	6	60	7	5.1	3.6**	129	2.6	0.9	8.9
Senegal	47	3	97	0.8	1.1	395	0.2	2.2	0.65
Sierra Leone	37	0	100	1.8	1.6#	440	0.4	2.5	1.4
Somalia	28	96	3	0.7	1.6#	148	0.4	1.1	1.3
South Africa	57	15	0	21.5	1.3	3.5	17.0	0.028	62.1
Sudan	36	42	56	2.3	1.6#	281	0.7	1.7	2.5
Swaziland	26	69	0	38.8	1.6#	21	26.1	0.17	107.0
Tanzania	32	21	75	8.8	2.7	372	2.0	2.1	7.4
Togo	33	0	100	4.1	2.6	445	0.9	2.5	3.0
Uganda	14	20	73	4.1	1.9	437	1.1	2.6	4.1
Zambia	40	16	83	16.5	2.1	396	3.6	2.3	14.0
Zimbabwe	35	54	0	24.6	1.2	16	16.7	0.128	61.9
Average††	34	23	67	6.9	2.0	320	1.3	1.8	4.9
Median	33	14	85	4.8	1.6	396	1.2	2.2	4.2

*Malaria incidence and deaths denote estimates in the absence of HIV, all age groups combined. Abbreviations: /1,000py = per 1,000 person-years. †Excluded from analysis were the following small countries: Lesotho and the islands The Seychelles, Reunion, Comoros and Mauritius, which are at negligible malaria risk, and Sao Tome & Principe and Cape Verde, which may be subject to stable malaria transmission but whose transmission intensity has not been precisely characterized in the Malaria Risk in Africa model. ‡Calculated by using country-specific age distributions. §Estimates by UNAIDS/World Health Organization (WHO) for end of 2003 (*28*). ¶From estimates by UNAIDS/WHO for end of 2003 or from national household surveys (*11*). Same ratios assumed for children as for adults. #In the absence of reliable urban/rural data, we applied the median urban/rural ratio across countries with urban and rural data. **In the absence of specific urban and rural data, the estimate for neighboring Burundi, which was judged to be similar in HIV epidemiology, was applied. ††Weighted according to population size, except for the increases in malaria incidence and deaths due to HIV-1, which are weighted according to the absolute number of malaria cases and deaths in each country.

In Botswana, Namibia, South Africa, Swaziland, and Zimbabwe, only areas with a climate suitability index >0.75 on the Malaria Risk in Africa map were considered to have transmission of unstable nature because vector control has been successful in some places (*19*). The average incidence in malarious areas in these countries has been estimated as 0.0294 per person per year (*19*). Age-specific incidence rates from clinic data have only been reported from Zimbabwe. We, therefore, applied the age distribution from areas of low transmission in Central Africa (see above and Table 1), which gave proportions of cases among children <5 years of age from 16% to 22%, in agreement with the 21% among reported cases in Zimbabwe.

We assumed that the urban-rural ratio in malaria incidence rates was 0.50, a conservative estimate based on 3- to 24-fold lower entomologic infection rates in periurban and urban areas compared to rural areas (*20*) and the approximately linear increase in infection rates with increasing entomologic infection rates up to a certain saturation point (*29*). Proportions of urban persons were assumed to be the same in areas of high, low, and unstable transmission, because the Malaria Risk in Africa risk classification (*18*) did not incorporate small-scale variation due to urban environment. Urban and rural populations were based on countries' definitions (*30*), without standardization between countries.

### Effects of HIV-1 on Malaria Incidence

The best evidence for associations between HIV-1 and clinical malaria comes from 2 longitudinal studies in Uganda, where malaria transmission is of high intensity. A community-based study in rural Masaka found odds ratios of clinical malaria among HIV-positive adults compared to HIV-negative adults, of 1.2, 3.4, and 6.0 for CD4 counts >500/μL, 200–499/μL, and <200/μL, respectively (p = 0.0002) (*9*). As in earlier studies, malaria incidence was defined as any acute fever concurrent with malaria parasitemia, without excluding alternative causes of the fever through further laboratory tests. Because malaria infection may occur without symptoms, in particular among adults in settings of high-intensity transmission, and because HIV-infected people often have acute nonmalarial fevers (*10*), the effect of HIV may have been overestimated.

The second study in Uganda, on HIV-positive persons only, excluded alternative causes of acute febrile illness, such as bacteremia (*10*). Malaria incidence for CD4 counts >500/μL, 200–499/μL, and <200/μL was 57, 93, and 140 per 1,000 person-years, respectively, and when confined to fever episodes with high parasitemia, 22, 53, and 90 per 1,000 person-years (*10*). We, therefore, assume that HIV-1 increases malaria incidence in adults by 1.2, 3, and 5 times for the above CD4 categories.

No community-level data are available for children, but 4 hospital-based cohorts in settings of high malaria transmission were studied in the late 1980s (*6**,**8**,**27**,**31*). In a birth cohort in Kinshasa, HIV-1 infection at any stage and clinically diagnosed AIDS increased malaria incidence by 1.2- and 2-fold, respectively, but the increases were not significant (*8*). A birth cohort in Blantyre, Malawi, found no HIV-related increase in the incidence of parasitemia (*31*). In Kampala, perinatally HIV-infected children with AIDS experienced notably fewer malaria episodes than HIV-uninfected children (*6*), which the authors attributed to the increased use of chloroquine before hospitalization among the HIV-infected children. In contrast, among children in Kinshasa from 1986 to 1988 who had received blood transfusions, those who were HIV-infected experienced 1.4 times more clinical malaria than those who were not (*27*). The effect of HIV-1 on malaria incidence is likely less apparent in children than in adults in high-transmission areas because young children have a high incidence of symptomatic malaria anyway (*19*). However, the observations may be confounded by prehospital use of antimalarial drugs or the shorter follow-up times and younger ages of HIV-infected children. We assumed that HIV-1 does not increase malaria incidence in children <5 years of age in high-transmission areas. Few data from areas of low-level and unstable malaria transmission in Africa exist and we assumed that HIV-1 increases malaria incidence equally in adults and children by the risk ratios specified above for adults in high-transmission areas.

The observed effect of HIV-1 on the incidence of clinical malaria may in part be the result of an increased incidence of recrudescences after failing antimalarial treatment because HIV-1 lowers the efficacy of antimalarial treatment (*32**,**33*). No published studies report specifically on the effect of HIV on malaria recrudescence. If such an effect exists, however, it will have been accounted for under the assumed overall effect of HIV on clinical malaria, since none of the studies from which we derived this assumed overall effect (*6**,**8**–**10**,**27**,**31*) adjusted malaria incidence rates observed in HIV-positive and HIV-negative participants for differences between these groups in treatment failure.

### Malaria Mortality and Effect of HIV

Malaria mortality was derived from malaria incidence, assuming fixed case-fatality rates. In high-transmission areas, 0.3% of malaria episodes were assumed to be fatal in adults and 0.8% in children (*21*). In areas of low-level transmission, we assumed a fatality rate of 0.8% for all ages (*21*).

Studies on adults in areas of high malaria transmission showed that HIV-1 infection increased case fatality among hospitalized persons with severe malaria by 1.6- to 2.5-fold (*7**,**34**,**35*), while the incidence of severe malaria, a precursor of fatal episodes, increased by 2.7-fold (*36*). In children exposed to high transmission in Kinshasa, HIV-1 increased the rate of hospitalization for malaria by 6-fold and malaria case fatality by 9.8-fold, although these effects were not significant; among HIV-infected persons who did not meet clinical criteria for AIDS, rates of hospitalization or death due to malaria did not increase (*8*). In Kampala, perinatally infected children were hospitalized for malaria 2.8 times more often than HIV-uninfected children (p = 0.001) (*6*).

In areas of low intensity and unstable malaria transmission, malaria diagnosis is less problematic because of less acquired immunity. Hospital-based studies in Zimbabwe in 1999 and Kwa-Zulu Natal, South Africa, in 2000, documented 6.9- and 8.8-fold increases in malaria case fatality among HIV-infected adults relative to HIV-negative patients (*4**,**5*). In Soweto, South Africa, a significant 1.7-fold increase in the rate of severe malaria was observed (*37*). For children living in areas of unstable malaria transmission, a hospital-based study in Kwa-Zulu Natal found a 2.7-fold increase in severe malaria (p = 0.05) and a 3.6-fold increase in fatality among severe cases (p = 0.1) associated with HIV-1 (*3*).

These data collectively suggest that HIV-1 increases malaria deaths by increasing the proportion of severe cases, case fatality among them, and the failure rate of antimalarial treatment (*32**,**33*). We conservatively assumed that the malaria death rate is increased by 4 times, taking into account the problem of attributing fevers to malaria. Lacking further evidence, we applied this increase to all age groups and malaria transmission intensities. One study found that the effect of HIV-1 was greater for CD4 <200/μL (*37*), and we assumed that malaria death rate increases with falling CD4 counts in the same way as malaria incidence, giving relative death risks among HIV-1–positive participants relative to HIV-1–negative ones of 2, 4, and 10 for CD4 >500 cells/μL, 200–499/μL, and <200/μL.

### HIV-1 Prevalence

Estimates of national HIV-1 prevalence among adults (>15 years of age), children <5 years of age, and children 5–14 years of age are available from the Joint United Nations Programme on HIV/AIDS (UNAIDS) for 2003 (*28*). To evaluate impact separately for urban and rural areas, which differ in malaria transmission intensity (*20*), urban-to-rural ratios in HIV-1 prevalence were estimated from national household surveys or antenatal clinic surveillance data (UNAIDS and [11]).

### CD4 Distributions among HIV-infected Persons

The distribution of CD4 counts among HIV-infected persons follows from the pattern of CD4 decline after initial infection and the trend in HIV-1 prevalence over preceding years (Appendix). Data from a variety of populations not receiving antiretroviral therapy suggest that CD4 decline is approximately linear after infection with HIV-1 (*26**,**38*). In African patients, CD4 declines from ≈825/μL, the median value in HIV-uninfected adults (*23**–**26**,**39*), to a mean of 20/μL at death of AIDS (*40*).

**Figure 1:**
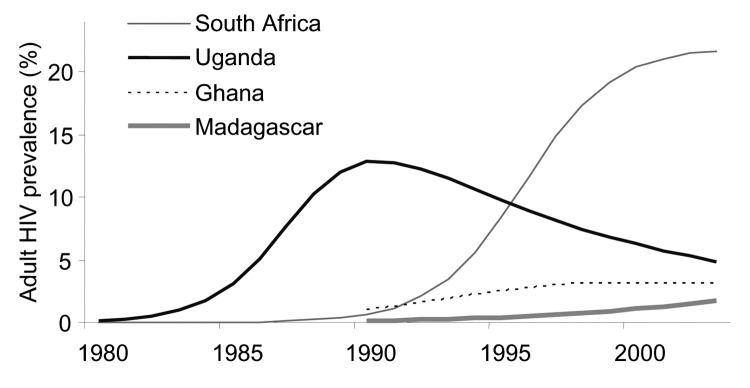
Modeled time trends in HIV-1 prevalence (adults 15–49 years), based on UNAIDS estimates from sentinel surveillance data in antenatal clinics (*28*).

**Figure 2:**
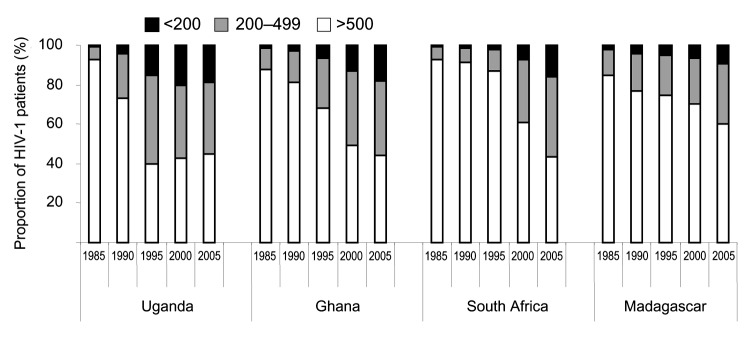
Modeled time trends in CD4 count distributions (per microliter) among HIV-infected adults in selected African countries. Madagascar: example of a rising HIV-1 epidemic at low grade; Ghana: example of a stable epidemic at low grade; Uganda: example of a high-grade epidemic that has declined and leveled off; South Africa: example of a high-grade epidemic that recently started leveling off.

CD4 distributions among HIV-infected adults in countries with different HIV-1 epidemics were determined by exploring 4 different epidemic patterns: 1) Uganda, where adult prevalence fell from a peak of ≈13% in the early 1990s to an estimated 4.1% (2.8%–6.6%) in 2003; 2) Ghana, where adult prevalence was relatively stable in recent years at 3.1% (1.9%–5.0%) in 2003; 3) South Africa, where the epidemic started only in the 1990s but reached an estimated prevalence of 21.5% (18.5%–24.9%] in 2003; and 4) Madagascar, where adult prevalence has risen rapidly in recent years to an estimated 1.7% (0.8%–2.7%) in 2003 (Figure 1) (*28*)]. In all 4 countries, most HIV-1 patients had CD4 >500/μL at the start of the epidemic (Figure 2). In Uganda, the prevalence of CD4 <500/μL rose rapidly until 1996, 6 years after the peak in HIV-1 prevalence. In the other epidemics, the prevalence of low CD4 rose more slowly, following their later stabilization. At means of 430 to 660/μL for 1996, the modeled CD4 counts are consistent with empiric data on HIV-infected African adults, which found means of 400 to 630/μL (*23**,**24**,**26*) and medians of 325 to 660/μL (*9**,**23**,**24**,**26**,**39*). Also, the modeled CD4 counts for South Africa from 2000 to 2005 (Figure 2) were in agreement with the observed distribution of 50%, 40%, and 10% with CD4 >500/μL, 200–499/μL, and <200/μL, respectively, in Soweto in 2002 (*39*).

Despite their different epidemic curves, the modeled CD4 distributions among HIV-1 patients were fairly similar for Uganda, Ghana, and South Africa in 2004: 44%–45% have CD4 >500/μL, 36%–41% have CD4 200–499/μL, and 15%–19% have CD4 <200/μL. We assume similar CD4 distributions for all other countries in which HIV prevalence has also stabilized. In Madagascar only, where HIV prevalence still increases rapidly, CD4 counts are notably higher (Figure 2). We therefore applied 2 distributions: in Madagascar, 60% of HIV-infected adults had CD4 >500/μL, 30% had CD4 200–499/μL, and 10% CD4 <200/μL; for other countries, assumed proportions were 44%, 39%, and 17%, respectively.

## Results

HIV-1 increased malaria incidence by 0.20% to 28% across countries (Table 2). The largest increases were in Botswana, South Africa, Swaziland, Zimbabwe, and Namibia, where HIV-1 prevalence is highest, especially in rural areas, and malaria transmission most unstable. For any given HIV-1 prevalence, HIV-1 impact was greatest in countries with low-intensity or unstable malaria transmission, where relatively more malaria occurs in adults. For example, impact was greater in Burundi than in Liberia, where malaria transmission is higher, despite an HIV prevalence of ≈6% in both countries. Outside southern Africa, impact was relatively high in the Central African Republic, with a comparatively high HIV-1 prevalence rurally, and Kenya, with a high proportion of low-intensity malaria transmission.

Across 41 countries, HIV-1 increased malaria incidence by 1.3%. This relatively small impact is explained, first, by the different geographic distributions of the 2 diseases. Malaria incidence rates are highest in West and Central Africa, where HIV-1 prevalence is comparatively low (Table 2). HIV-1 is most prevalent in southern Africa, where malaria transmission is rarely stable and comparatively well controlled. Second, within countries, the impact of HIV-1 was further limited because HIV-1 is more prevalent in cities (median urban/rural ratio 1.6, Table 2), whereas malaria is more prevalent rurally (assumed urban/rural ratio 0.5). Third, HIV-1 mainly affects adults, whereas in countries of high malaria transmission, malaria has the greatest impact on young children.

**Figure 3:**
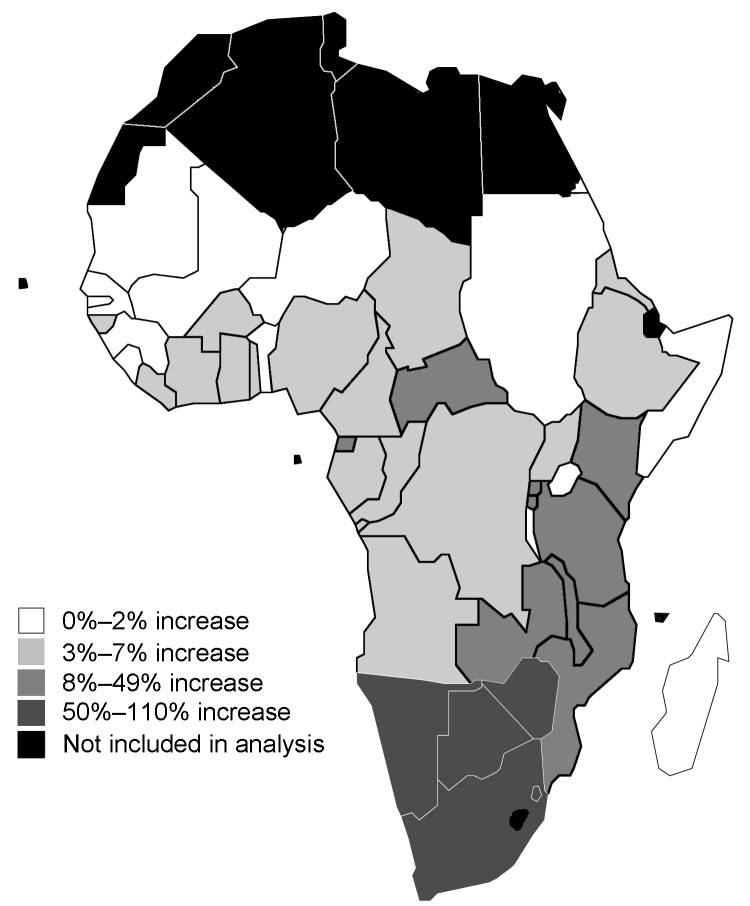
Estimated proportional increases in malaria deaths due to HIV-1 in sub-Saharan African countries in 2004, for all ages combined.

HIV-1 increased malaria deaths by 0.65% to 114% across countries (Table 2). As for malaria incidence, the largest increases were in southern Africa (Figure 3), and the ranking among countries was very similar to the ranking by impact on malaria incidence. On a continental scale, HIV-1 increased malaria deaths by 4.9%. The impact on malaria deaths was greater than that on malaria incidence for 2 reasons. First, in the individual patient, HIV-1 increases death risk more than incidence (Table 1). Second, the impact of HIV-1 on death was compounded by its impact on incidence rates.

Against our baseline of ≈228 million malaria cases and ≈1.3 million malaria deaths among all ages in sub-Saharan Africa, these increases would correspond to an additional 3 million malaria cases and 65,000 malaria deaths annually due to HIV-1, or to ≈3% of the estimated 2.3 million HIV/AIDS deaths in sub-Saharan Africa in 2004 (*28*). However, the assumed baseline malaria incidence and death rates were cruder than we would have liked and do not take into account the impact of malaria control in the different countries. We, therefore, focus on relative increases in malaria due to HIV.

### Alternative Scenarios

To assess the sensitivity of results to assumptions made, we recalculated the impact of HIV-1 for several alternative scenarios (Table 3). Estimated impact increased or decreased considerably with larger or smaller assumed relative risks at the individual level. Most critical, however, were the assumed age patterns in malaria incidence and case fatality. The smaller the decline with age in malaria incidence and fatality rates, the greater the impact of HIV-1 would be (<12.5% increase in malaria deaths and 4.4% increase in malaria incidence, Table 3) because of the concentration of HIV-1 in adults.

**Table 3:** Univariate sensitivity analyses of HIV-1 impact on malaria incidence and deaths, sub-Saharan Africa, 2004*

Scenario	% increase in malaria incidence due to HIV (minimum and maximum)†	% increase in malaria deaths due to HIV (minimum and maximum)†
Default scenario: see Tables 1 and 2.	1.3 (0.20–28)†	4.9 (0.65–114)†
Weaker effect of HIV-1 on malaria incidence: RR = 1.0 at CD4 >500/μL, RR = 2.0 at CD4 200–499/μL, and RR = 4.0 at CD4 <200/μL.	0.8 (0.11–16)	4.4 (0.60–90)
Stronger effect of HIV-1 on malaria incidence: RR = 1.5 at CD4 >500/μL, RR = 4.0 at CD4 200–499/μL, and RR = 8.0 at CD4 <200/μL.	2.2 (0.33–47)	5.7 (0.73–153)
Weaker effect of HIV-1 on malaria mortality (all groups): RR = 1.5 at CD4 >500/μL, RR = 2.0 at CD4 200–499/μL, and RR = 4.0 at CD4 <200/μL.	n.a.	2.4 (0.30–59)‡
Weaker effect of HIV-1 on mortality in children <5 y in areas of high malaria transmission specifically, analogous to the comparatively weak effect of HIV-1 on incidence in this group: RR = 1.5 at CD4 >500/μL, RR = 2.0 at CD4 200–499/μL, and RR = 5.0 at CD4 <200/μL.	n.a.	3.7 (0.45–114)
Stronger effect of HIV-1 on malaria mortality (all groups): RR = 3.0 at CD4 >500/μL, RR = 6.0 at CD4 200–499/μL, RR = 12.0 at CD4 <200/μL.	n.a.	6.9 (0.92–157)§
Stronger decrease with age in malaria incidence: RRs compared to <5 y of 0.30 for 5–14y and 0.05 for >15 y in high malaria transmission areas, and 0.60 for 5–14 y and 0.10 for >15 y for areas of low and unstable malaria transmission including southern African countries.	1.0 (0.13–15)	4.0 (0.45– 59)
No decrease with age in malaria incidence at any malaria transmission intensity.	4.4 (0.54–37)	12.5 (1.5–153)
Stronger decrease with age in malaria CFR: 1.2% in <5 y at all malaria transmission intensities, 0.8% in >5 y at low and unstable transmission, 0.15% in >5 y at high transmission.	n.a.	4.0 (0.53–107)
No decrease with age in malaria CFR at any transmission intensity.	n.a.	5.7 (0.77–114)
HIV-1 increases malaria incidence also in children <5 y in areas of high malaria transmission.	2.0 (0.26–28)	5.9 (0.78–114)
CD4 count decline during HIV-1 infection: 1,000–100/μL¶	1.0 (0.15–21)	3.7 (0.50–82)
CD4 count decline during HIV-1 infection: 700–0/μL#	1.7 (0.24–35)	6.2 (0.81–151)
No urban/rural difference in the malaria incidence rate	1.4 (0.20–28)	5.3 (0.67–114)
Lower HIV prevalence in adults: lower bound country estimates by UNAIDS/WHO**	0.9 (0.08–27)	3.2 (0.28–108)
Higher HIV prevalence in adults: upper bound country estimates by UNAIDS/WHO**	2.2 (0.37–29)	8.0 (1.2–121)
Lower HIV prevalence in children <14 y: lower bound country estimates by UNAIDS/WHO††	1.3 (0.20–27)	3.9 (0.47–111)
Higher HIV prevalence in children <14 y: upper bound country estimates by UNAIDS/WHO††	1.5 (0.23–29)	6.8 (1.08–119)

*N.A., not applicable; CFR, malaria case-fatality rate; RR, relative risk; UNAIDS, Joint United Nations Programme on HIV/AIDS/WHO. †Continental total. In none of the scenarios did the ranking of countries in magnitude of HIV impact change appreciably. Across all scenarios, the minimum and maximum increases (in brackets) were always in Senegal or Mauritania, and in Botswana, respectively. An exception was the scenarios of lower HIV prevalence in adults, for which the lowest malaria impacts would be in Sierra Leone and Somalia. ‡The overall relative risk for malaria mortality due to HIV-1 in stable HIV-1 epidemics is now 2.1, i.e., does no longer fit the observed value of ≈4 (see Methods, Malaria mortality and Effect of HIV). §The overall relative risk for malaria mortality due to HIV-1 in stable HIV-1 epidemics is now 5.7, i.e., does no longer fit the observed value of ≈4 (see Methods, Malaria death and effect of HIV). ¶As in Western populations (*25*). #To allow for a possible initial drop in CD4 immediately upon infection, i.e., still before seroconversion. **Cross-country median lowerbound estimate of HIV prevalence in adults 2.7%; cross-country median upperbound HIV prevalence estimate 8.8% (compared to default point estimate of 4.8%). ††Cross-country median lowerbound estimate of HIV prevalence in children <14 years of 0.2%; cross-country median upperbound HIV prevalence estimate 1.1% (compared to default point estimate of 0.5%).

Estimates were relatively insensitive to whether the effect of HIV-1 on malaria incidence applied also to children in high-transmission areas, as a recent study in Uganda suggested (*41*). Alternative assumptions concerning the range over which CD4 counts decline during HIV infection also made little difference. Abandoning the assumption that malaria occurs more frequently in rural than in urban areas resulted in only a slight increase in HIV-1 impact because the countries with highest HIV-1 prevalence and Nigeria, which has most malaria cases, had similar HIV-1 prevalence in cities and rural areas (Table 2). Finally, estimated impacts were moderately sensitive to uncertainties in national HIV prevalence for adults but not to uncertainties in HIV prevalence for children.

Combining the ranges of estimates from these scenarios into 1 multivariate analysis, with the Monte Carlo technique and assuming triangular distributions for all parameters, overall 95% confidence intervals (CIs) on the continentwide estimates would be 0.6%–7.9% (best estimate ≈1.3%) for clinical malaria incidence, and 3.1%–17.1% (best estimate ≈4.9%) for malaria deaths. For Botswana, the country with the largest estimated HIV impact, 95% CI would be 14%–47% (best estimate ≈28%) for malaria incidence and 37%–188% (best estimate 114%) for malaria deaths.

## Discussion

Across 41 countries in sub-Saharan Africa, the HIV-1 epidemic may have increased the incidence of clinical malaria by 1.3% (95% CI 0.6%–7.9%) and malaria deaths by 4.9% (95% CI 3.1%–17.1%) in 2004. Continentwide impact was limited by the different geographic distributions of the 2 diseases and their different age patterns.

For southern Africa, estimated proportional increases were <28% (95% CI 14%–47%) for malaria incidence and <114% (95% CI 37%–188%) for malaria deaths. An impact of HIV-1 of this magnitude may have contributed to observed increases of malaria in the 1990s in areas of unstable transmission, including Kwa-Zulu Natal (*13**,**14*) and northern Zambia (*15*). Outside southern Africa, however, HIV-1 is unlikely to be a major contributor to rises in malaria, and where this appears to be so, a more plausible explanation may be overdiagnosis of fevers as malaria in HIV-1 patients. Such over-diagnosis may occur unintentionally in settings where malaria is diagnosed without parasitologic confirmation because of the increased frequency of acute fevers in HIV-1 patients (*10*). Intentional misdiagnosis could also occur, if doctors are reluctant to diagnose illness as HIV-related for fear of social stigma.

These estimates have several limitations. First, the magnitude of effects of HIV-1 on malaria incidence and death risk in individual patients is critical (Table 3) but uncertain because of diagnostic problems in settings of high malaria transmission and a lack of population-based data from areas of low intensity and unstable transmission.

Second, results are sensitive to age patterns in malaria (Table 3), which are not well known. The sharp contrast in estimated impact of HIV-1 between the 5 southern African countries and the remainder of Africa depends on the assumption that malaria declines more slowly with age in South Africa, where all malaria is assumed to be unstable. In practice, the shift from unstable to stable malaria transmission, i.e., from clinical effects in all age groups to a predominance in young children, is more gradual; thus, effects of HIV on malaria in Zimbabwe and Zambia, for example, may be more similar than we estimated. The estimation method developed here could, nevertheless, be applied to more refined age-specific estimates of malaria incidence and death.

Finally, subnational heterogeneity in malaria or HIV, apart from urban/rural differences, was not considered, and this fact may have biased the estimation for countries where either or both diseases are heterogeneously distributed, such as Kenya, Ethiopia, Tanzania, and South Africa (*42*). For example, in South Africa, both malaria and HIV-1 are concentrated in Kwa-Zulu Natal, so that their interaction may be greater than our estimate.

The impact of HIV-1 that we have estimated only pertains to malaria cases and deaths and does not include effects on anemia or adverse birth outcomes attributable to concurrent malaria and HIV-1 in pregnant women. In areas of high-intensity transmission, such as in Kenya and Malawi, the latter effects might be more important than malaria cases and deaths per se. Also, our analysis did not cover the effect of HIV-1 on demand for antimalarial drugs. In most of rural Africa, antimalarial drugs are presumptively prescribed to treat any fever without an obvious nonmalarial cause. Recurrent fevers in HIV-1 patients may, therefore, cause considerable overuse of antimalarial drugs, increasing not only costs but also the risk for drug resistance. The HIV-1 epidemic thus underlines the need to improve capacity for laboratory diagnosis of febrile disease in Africa.

To limit the impact of HIV-1 on malaria, HIV-infected persons, in addition to young children and pregnant women, may form a target group for provision of insecticide-treated mosquito nets (*2*). In areas of low intensity and unstable transmission, HIV may be a reason for intensifying or resuming indoor residual spraying to control malaria vectors. For HIV-infected persons who are prone to treatment failure with conventional antimalarial drugs (*27**,**32**,**33**,**43*), effective combination therapy is of utmost importance.

Highly active antiretroviral combination therapy has great potential to reduce HIV-related malaria (*44*). Cotrimoxazole prophylaxis, recommended for adults and children living with HIV in Africa (*45*), is also effective in reducing clinical malaria, independent of baseline CD4 (*41**,**46**,**47*). Combined HIV and malaria interventions might best be delivered at peripheral health centers, including antenatal clinics (*2*).

HIV-1 appears to have increased the impact of malaria disease and death in South Africa compared to the 1980s, although data do not allow a precise quantification of this effect. In areas of high HIV and low-intensity or unstable malaria, continued vigilance and intensified malaria control are indicated. In HIV-infected adults, pregnant women, and children, malaria is among the simplest opportunistic infections to prevent and treat.

## Appendix

### Estimating Distribution of CD4 Cell Counts among HIV Patients over the Course of the Epidemic

When HIV is first introduced into a population, most of the infected persons must have been recently infected and still have high CD4 cell counts. The CD4 cell count distribution among HIV-positive persons later in the epidemic is determined by, first, the frequency distribution of the times since infection among HIV-positive people at that time, and second, the frequency distribution of CD4 cell counts among HIV-positive people at different times since infection.

We estimate the frequency distribution of the times since infection by modeling HIV incidence and deaths from its prevalence, as a function of calendar time. Trends over time in prevalence of HIV infection were estimated by fitting double logistic curves to the data of the prevalence of HIV among women attending antenatal clinics. To convert prevalence to incidence, we assumed that the survival distribution of HIV-1 infected adults is a Weibull function with median of 9.0 years (*48*) and a shape parameter of 2.28 (*49*).

If no one died of AIDS, the incidence, *I*_0_(*t*), would be equal to the time derivative of the prevalence curve, *P*(*t*), so that



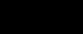

 1


To correct for the effect of AIDS-related mortality, if the proportion of persons who survive for *t* years after infection is *W*(*t*), the probability that persons die *t* years after they are infected is



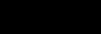

 2


Since deaths will decrease prevalence, we add deaths due to all previous incident infections to our estimate of incidence so that



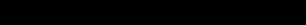

 3


Equation 3 can be solved using Fourier transforms (indicated with curly-brackets) so that



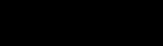

 4


The probability that a person who is alive and HIV positive at the present time *t* was infected with HIV at time *t* − τ is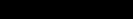 5enabling us to calculate the probability density of times since infection as a function of calendar time.

For the density function of CD4 cell counts as a function of time since HIV-1 infection, data from a variety of populations not receiving antiretroviral therapy suggest that the decline in CD4 counts is approximately linear throughout the survival with HIV-1 (*49**,**50*). If the CD4 cell counts, *c*, start from a nominal value of 1 and if τ is the time of death, the expected value of *c* is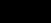 6where the probability density function of τ is
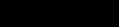
 7and



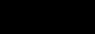

 8


Equation 8 is easily evaluated analytically. Scaling the CD4 cell decline curve to observed mean cell counts (in this study, 825 cells/μL at seroconversion, the median value found in African HIV-1 uninfected adults [50-54], and 20/μL at death from AIDS [55]), this gives the probability density function of the CD4 cell counts as a function of time since infection for different calendar times.
